# Lateralization of bladder function in normal female canines

**DOI:** 10.1371/journal.pone.0264382

**Published:** 2022-03-01

**Authors:** Dania Giaddui, Danielle S. Porreca, Ekta Tiwari, Nagat A. Frara, Lucas J. Hobson, Mary F. Barbe, Alan S. Braverman, Justin M. Brown, Michel A. Pontari, Michael R. Ruggieri Sr.

**Affiliations:** 1 Department of Cardiovascular Sciences and Center for Translational Medicine, Lewis Katz School of Medicine, Temple University, Philadelphia, Pennsylvania, United States of America; 2 Department of Neurosurgery, Neurosurgery Paralysis Center, Massachusetts General Hospital, Boston, Massachusetts, United States of America; 3 Department of Urology, Lewis Katz School of Medicine, Temple University, Philadelphia, Pennsylvania, United States of America; 4 Shriners Hospitals for Children of Philadelphia, Pennsylvania, Philadelphia, United States of America; University Medical Center Utrecht, NETHERLANDS

## Abstract

This study aimed to identify potential lateralization of bladder function. Electrical stimulation of spinal roots or the pelvic nerve’s anterior vesical branch was performed bilaterally in female dogs. The percent difference between the left and right stimulation-induced increased detrusor pressure was determined. Bladders were considered left or right-sided if differences were greater or less than 25% or 10%. Based on differences of 25%, upon stimulation of spinal roots, bladders were left-sided in 17/44 (38.6%), right-sided in 12/44 (27.2%) and bilateral in 15/44 (34.2%). Using ± 10%, 48% had left side dominance (n = 21/44), 39% had right side dominance (n = 17/44), and 14% were bilateral (n = 6/44). With stimulation of the pelvic nerve’s anterior vesical branch in 19 dogs, bladders were left-sided in 8 (42.1%), right-sided in 6 (31.6%) and bilateral in 5 (26.3%) using 25% differences and left side dominance in 8 (43%), right sided in 7 (37%) and bilateral in 4 (21%) using 10% differences. These data suggest lateralization of innervation of the female dog bladder with left- and right-sided lateralization occurring at similar rates. Lateralization often varied at different spinal cord levels within the same animal.

## Introduction

Lateralization, or the phenomenon by which the nervous system organizes itself in an asymmetric fashion, has been an emphasis of study across vertebrate species. Behavioral lateralization is evident in dogs in a variety of functions including paw preference, asymmetric tail wagging, visual processing, and auditory processing [1]. In addition, functional cerebral asymmetry has been recently demonstrated in dogs in which either the left or right brain hemispheres is responsible for certain motor functions [2]. Although lateralization has been identified in dogs [3], less is known about the lateralization of function of visceral organs such as the bladder.

Regulation of the physiological activities of the urinary bladder requires coordination between extensive neuronal circuitry within the brain and spinal cord, and the network of peripheral neurons that directly interface with the bladder [4]. Both left and right sacral and lumbar spinal cord segments contain preganglionic neurons of parasympathetic and sympathetic origin. In regard to the innervation of lower urinary tract smooth muscle, parasympathetic innervation is responsible for synchronous detrusor contraction and urethral relaxation to allow for micturition, while sympathetic innervation is responsible for synchronous detrusor relaxation and urethral contraction to store urine [5].

This study is part of a larger investigation that aims to establish surgical reinnervation of the dog bladder following long-term lower spinal root injury via bilateral transfer of the obturator nerve branches and redundant branches of the sciatic nerve [6–10]. Thus, if one had to choose one side to reinnervate for return of bladder function, is the left side or the right side a better choice? Our group previously noted an incidental observation of left-dominant laterality in dog bladder responsiveness to pelvic nerve stimulation in 4 of 8 animals, right side dominance in 2 and equivalent responsiveness in 2 [11], although innervation lateralization of the dog bladder was not the emphasis of these past investigations. Previous surgical trials in humans by one group to establish an artificial somatic-autonomic reflex arc for restoration of bladder and bowel functions used exclusively the left side [12, 13].

Here, we specifically address the contributions of the left and right spinal cord segments (L6, L7, S1, S2, and S3) and anterior vesical branches of the pelvic nerves to bladder function in a larger cohort of dogs. Using electrophysiology studies, we unilaterally stimulated the left or right spinal roots, or the pelvic nerve’s anterior vesical branch, to assess their relative contribution to bladder contractility. Understanding the patterns of laterality in our model may be useful when interpreting results of our nerve transfer reinnervation procedures [6–10, 14–16]. It could also be an important factor to consider for pelvic organ reinnervation in humans.

## Methods

### Animals

Data from 57 total female mixed-breed mongrel hound dogs, 6–12 months with an average weight of 16.7–25 Kg (Marshall BioResources, North Rose, NY) were used. All studies were approved by the Animal Care and Use Committee at Temple University (Philadelphia, PA; Protocol #4480) and were in accordance with guidelines of both the National Institute of Health guidelines and the United States Department of Agriculture and American Association for Assessment of Laboratory Animal Care. Animals were group housed on a 12-hour light/dark cycle with environmental enrichment toys and unlimited access to food and water.

### Spinal root & pelvic nerve’s anterior vesical branch functional electrical stimulation

Prior to surgery, animals received 6 mg/kg IV of propofol to allow for the insertion of the endotracheal tube. Anesthesia was then maintained using isoflurane at 2–3% maximum alveolar concentration with oxygen. All animals underwent lumbosacral laminectomy of L6-S3 vertebrae to expose the lower spinal cord and spinal roots, and to identify nerves via electrical stimulation, as previously described [11, 16]. Bladder pressure monitoring was obtained by catheterizing the urethra with a double balloon catheter connected to external pressure transducers for the continuous recording of bladder and urethral pressures and for bladder filling with a syringe pump. The double balloon catheter with a lumen at the end was positioned such that the balloon closest to the catheter end was positioned in the bladder neck to occlude the outlet and the adjacent balloon was positioned in the urethra such that the pressure inside the balloon was continuously monitoring urethral pressure. Balloon catheters were also inserted into the rectum at the level of the bladder dome interfaced with pressure transducers for the continuous recording of rectal pressures. The pressure transducers used were Becton Dickinson Dtxplus Model DT-XX with a 50 Hz low pass filter and a sampling rate of 4/second which were calibrated immediately prior to each procedure. The bladder capacity (defined as the volume inducing a marked increase in the slope of the volume-pressure curve) was determined with 3 successive filling cystometrograms using normal saline (30 mL/min) and the bladder was then emptied and filled to half of its capacity. Because of the urethral catheter, the bladder was not able to empty during the stimulations and thus isometric bladder contractions were recorded. The time between setting the bladder volume at half of its capacity and the stimulations were fairly consistent between different animals such that the bladder volume was relatively consistent between different animals.

**Fig 1:**
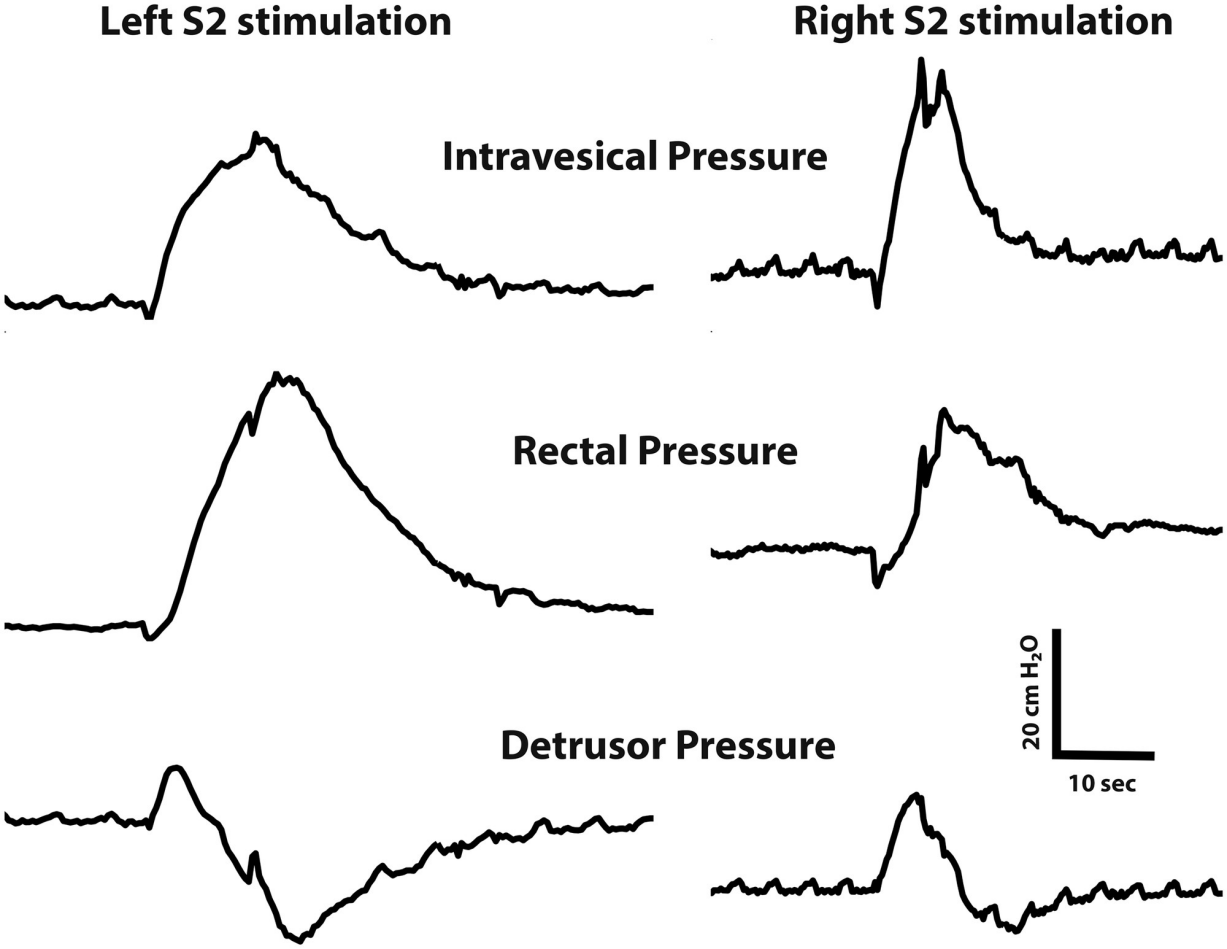
Left and right pressure recording of S2 spinal root stimulation. Representative example of a recording of intravesical, rectal and detrusor pressure during stimulation of the S2 spinal root. Pressure is measured in cm H_2_O.

Intraoperative electrical stimulation of left and right L6 through S3 spinal roots, or nerves within the pelvic plexus of the bladder, specifically the anterior vesical branch of the pelvic nerve emerging from the pelvic plexus towards the bladder body, was performed. Handheld monopolar or bipolar electrodes (tip diameter 0.5mm, Medtronic Xomed Inc.) with currents of 0.5–10 mA, a frequency of 20-Hz, and a pulse duration of 0.2 msec were used. These electrical stimulations were generated using a Grass Astro-Med model S88X stimulator interfaced stimulus isolation unit (SIU-C) for constant current square pulse trains of 2–5 second durations. If a monopolar electrode was used, the ground was connected over the chest skin. Changes in detrusor pressure (defined as rectal pressure subtracted from intravesical pressure) were continuously recorded with a PowerLab multichannel data acquisition system and LabChart software (ADInstruments, Colorado Springs, CO). Strength of nerve-evoked bladder contractions resulting from electrical stimulations were derived from differences between the resting baseline pressure and the peak pressure obtained during continuous stimulation. For stimulation of the spinal roots during which the abdomen was closed, detrusor pressure (intravesical pressure minus rectal pressure) is reported whereas during stimulation of the pelvic plexus during which the abdomen was open, intravesical pressure is reported. Intravesical, rectal and detrusor pressure recording for both the left and the right side of S2 spinal root stimulation are shown in Fig 1. To assure that fatigue of the detrusor muscle was not induced, multiple stimulations were separated by an average rest period of 1–2 minutes between stimulations. Data were included from animals which had both left and right side stimulations and the order of left versus right stimulation was not controlled. No criteria were used to exclude animals.

Each the 57 animals in the study underwent at least 1 of the stimulation protocols (spinal root(s) or pelvic nerve branch). Forty-four of these underwent stimulation of least one pair (right and left) of L6-S3 spinal roots, with right and left roots stimulated separately. Segmentally, right and left spinal roots were tested at L6 in 12/44 animals, at L7 in 28/44 animals, at S1 in 36/44 animals, at S2 in 32/44 animals, and at S3 in 18/44 animals. Thirty-five of the 44 animals received stimulations at multiple levels. Lastly, the right and left anterior vesical branch of the pelvic nerves were stimulated in 19 animals, 6 of which were part of the cohort of 35 animals tested at multiple levels.

One day prior to surgery, cephalexin (30–50 mg/kg) was administered orally twice per day and continued for 5 days post operatively. During the surgical procedures, vital signs and depth of anesthesia was continuously monitored and documented every 15 minutes including blood pressure, heart rate, respiratory pattern, arterial oxygen saturation body temperature, eyelid reflex, and withdrawal reflex (e.g. toe pinch). During the surgery, intravenous dopamine (7–12 μg/kg/hr) was administered if blood pressure decreased below the normal range. Prior to making the skin incision bupivacaine (0.5%, 10 mls) was administered subcutaneously along the incision site to prevent surgical wind-up. Acepromazine (0.025–0.2 mg/kg, subcutaneous) was administered after the animal emerged from anesthesia to allow calm and comfortable recovery. Immediately after surgery Buprenorphine (5–20 μg/kg) was administered subcutaneously and continued twice per day for a minimum of 2 days post operatively. Post- operatively body temperature, respiratory rate and capillary refill rate was monitored every 15 minutes until all parameters returned to normal values.

### Statistical analysis

**Fig 2:**
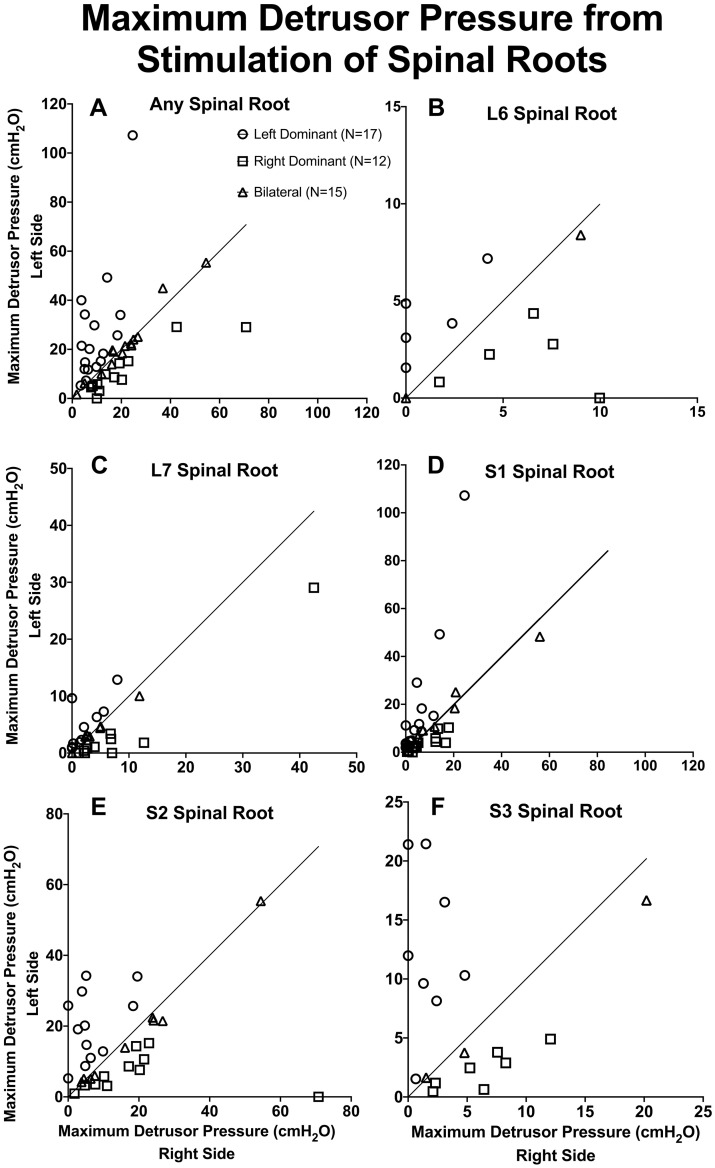
Maximum detrusor pressure after spinal root stimulation. A) Maximal contractions produced from electrical stimulation of L6 through S3 spinal roots were recorded during the initial surgeries in 44 of the 57 dogs. Left root- and right root-evoked contractions are displayed on the Y-axis and the X-axis, respectively. Each point represents the maximal contraction obtained from stimulation of either left or right spinal root in a single animal, whichever produced the strongest bladder contraction. B). Maximum detrusor pressure upon left and right L6 spinal root stimulations obtained from 12/44 dogs. C). Maximum detrusor pressure upon left and right L7 spinal root stimulation obtained from 28/44 dogs. D). Maximum detrusor pressure upon left and right S1 spinal root stimulation obtained from 36/44 dogs. E). Maximum detrusor pressure upon left and right S2 spinal root stimulation obtained from 32/44 dogs. F). Maximum detrusor pressure upon left and right S3 spinal root stimulation obtained from 18/44 dogs. L = Lumbar, S = Sacral, N = number of dogs, cmH_2_O = centimeter of water.

**Fig 3:**
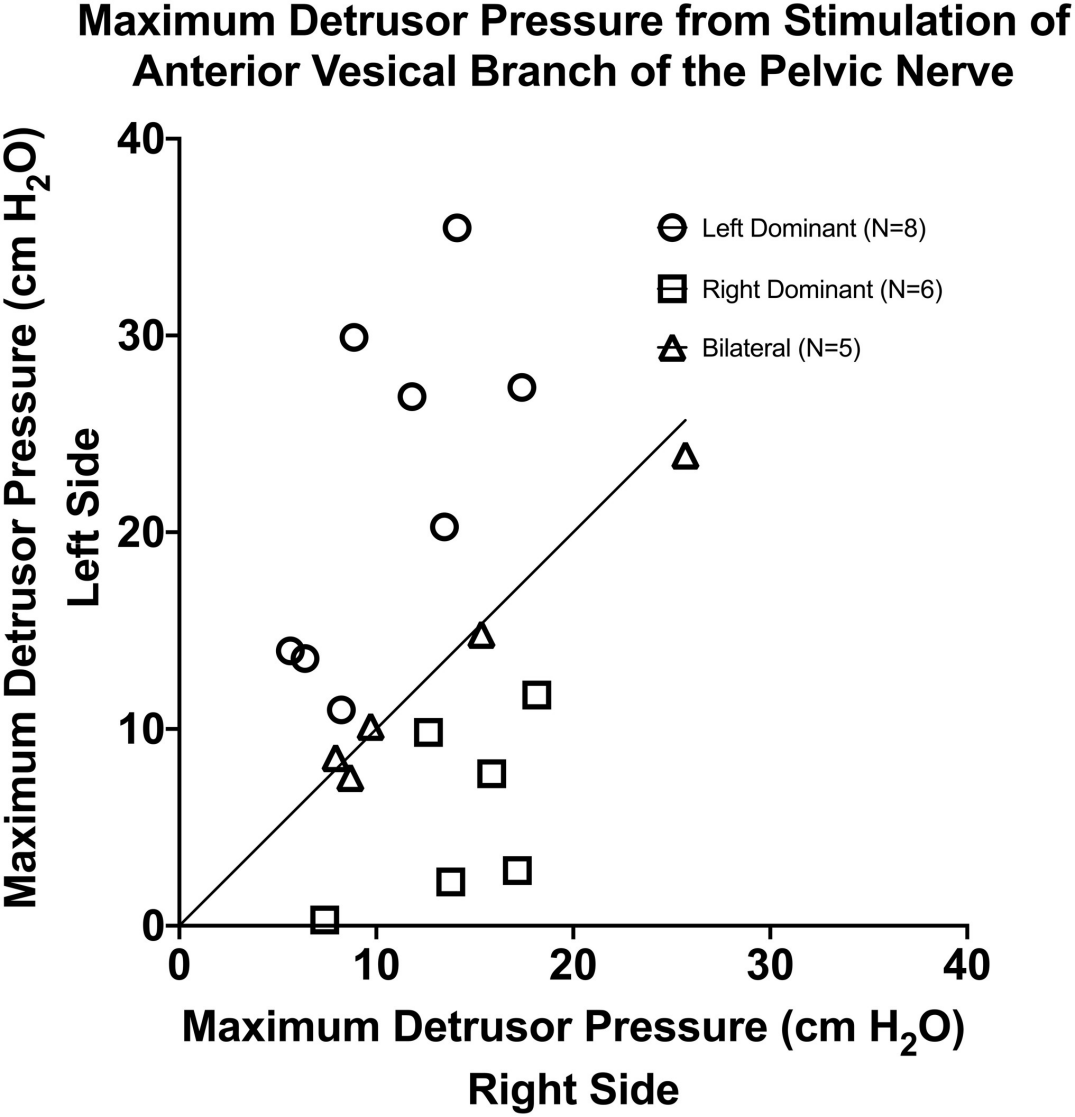
Maximum detrusor pressure after anterior vesical branch of the pelvic nerve stimulation. Maximal contractions produced from electrical stimulation of the anterior vesical branch of the pelvic nerve roots were recorded during the initial surgeries in 19 dogs (6 of which were part of the cohort tested at multiple levels). Left root- and right root-evoked contractions are displayed on the Y-axis and the X-axis, respectively. Each point represents the maximal contraction obtained from stimulation of either left or right pelvic nerve root in a single animal, whichever produced the strongest bladder contraction. N = number of dogs, cmH_2_O = centimeter of water.

The dominant side for functional innervation of the bladder in each animal was determined by calculating the percent difference between the left and right spinal root stimulation at spinal levels known to contribute to bladder function, as well as that of left and right anterior vesical branch of the pelvic nerve. We evaluated for left- or right-sided functional lateralization using two different parameters: either 25% or 10% difference when comparing sides. If differences were within the defined range, bladders were considered as bilaterally innervated functionally. The percent difference was calculated by taking the difference between left and right stimulations for each spinal root or pelvic nerve and dividing it by the average of both stimulations. For Figs 2A–2F and 3, the maximum detrusor pressure of the right side was plotted on the x-axis. Based on the percent differences, the maximum detrusor pressure of the left side was allocated to either left, right or bilateral on the y-axis with different symbols as shown on the Figures. All data were plotted along with a line of equivalence (y = x). Spearman and Pearson correlation tests (one tailed and two tailed) were performed using GraphPad Prism 8.4 (La Jolla, CA) for each spinal root and for pelvic nerve stimulation. A p-value of < 0.05 was considered to be statistically significant.

## Results

### Laterality determined from left and right stimulation of spinal roots

We examined detrusor contraction during electrical stimulation of spinal roots from L6-S3 vertebral levels in 44 dogs. Fig 2 shows the maximum detrusor pressure obtained after left or right stimulation of all spinal roots from L6-S3 combined in 44 animals (Fig 2A). The percent difference between left and right maximum detrusor pressure for each animal was calculated. 39% of the animals had left side dominance (n = 17, percent difference above 25%), 27% of the animals had right side dominance (n = 12, percent difference below -25%), and the remaining 34% of the animals were bilateral (n = 15, left and right max stimulations within ± 25% of each other, Fig 2A).

When the percent differences were redefined using ± 10%, 48% of the animals had left side dominance (n = 21, percent difference above 10%), 39% of the animals had right side dominance (n = 17, percent difference below -10%), and the remaining 14% of the animals were bilateral (n = 6, left and right max stimulations within ± 10% of each other). This is summarized in Table 1.

**Table 1:** Redefining lateralization as 10% difference between left and right spinal root (L6-S3) or anterior visceral branch of the pelvic nerve (upon stimulation).

Root/ PN Ant Br	Animals Tested	Left Dominance	Right Dominance	Bilateral
L6 Spinal Root	12	41.7%	41.7%	16.7%
L7 Spinal Root	28	42.9%	42.9%	14.3%
S1 Spinal Root	36	52.8%	47.2%	0%
S2 Spinal Root	32	43.7%	50.0%	6.3%
S3 Spinal Root	18	44.4%	50.0%	5.6%
Any Spinal Root	44	47.7%	36.8%	13.6%
PN Ant Br	19	42.1%	36.8%	21.1%

L = Lumbar, S = Sacral, PN Ant Br = Pelvic Nerve’s Anterior Vesical Branch

Differences between left and right maximum detrusor pressure were also evaluated at each individual spinal level (L6-S3), as shown in Fig 2B–2F. Upon L6 spinal root stimulation in 12 dogs, 5 of the bladders were left dominant (42%), 5 right dominant (42%), and 2 bilateral (17%, Fig 2B). L7 spinal root stimulation showed that out of 28 animals tested, 10 had left sided bladders (36%), 10 had right sided (36%), and 8 were bilateral (29%, Fig 2C). Stimulation of S1 spinal root in 36 animals showed that 16 animals were left dominant (44%), 14 were right dominant (39%), and 6 were bilateral (17%, Fig 2D). Similarly, S2 root stimulation indicated that out of 32 dogs, 12 were left side dominant (38%), 11 right side dominant (34%), and 9 were bilateral (28%, Fig 2E). For the 18 dogs tested at S3 spinal root, 8 were shown to have left side dominant bladder (44%), 7 right side dominant bladder (39%), and 3 were bilateral (17%, Fig 2F). Changes in the totals of each bladder type per spinal cord level when redefining lateralization as 10% difference between left and right are included in Table 1.

Of the 35 animals that received spinal root stimulations at multiple segmental levels, rarely did laterality occur exclusively on one side across all spinal levels and the anterior vesical branch of the pelvic nerve, as shown in Table 2.

**Table 2:** Lateralization of 35 dogs that underwent multiple segmental testing, based on the 25% and 10% differences between left and right stimulated spinal roots (L6-S3) and the pelvic nerve’s anterior vesical branch.

Animal Number	Based on 25% Difference						Based on 10% Difference					
	L6	L7	S1	S2	S3	PN Ant Br	L6	L7	S1	S2	S3	PN Ant Br
**1**			Lt	Lt	Lt	Lt			Lt	Lt	Lt	Lt
**2**	R	R	R	B	Lt		R	R	R	B	Lt	
**3**	R	R	B	B	B		R	R	R	B	R	
**4**	Lt	Lt	Lt	Lt	R	R	Lt	Lt	Lt	Lt	R	R
**5**	Lt	Lt	R	B	Lt	Lt	Lt	Lt	R	R	Lt	Lt
**6**			R	Lt					R	Lt		
**7**		B				B		R				B
**8**		R	Lt	B				R	Lt	R		
**9**		R	R	Lt				R	R	Lt		
**10**			R	Lt					R	Lt		
**11**		Lt	Lt	Lt		R		Lt	Lt	Lt		R
**12**		B	B	B	R			Lt	Lt	R	R	
**13**			B		R				Lt		R	
**14**			B	Lt		Lt			R	Lt		Lt
**15**	Lt	Lt	Lt	B			Lt	Lt	Lt	R		
**16**	B	B	Lt	Lt	Lt		B	B	Lt	Lt	Lt	
**17**	R	B					R	B				
**18**	Lt	R	R	Lt			Lt	R	R	Lt		
**19**	R	B	R	R			R	Lt	R	R		
**20**		R	Lt	Lt				R	Lt	Lt		
**21**	Lt	R	B	R			Lt	R	Lt	R		
**22**		Lt	Lt	Lt				Lt	Lt	Lt		
**23**	R	R	R	R			R	R	R	R		
**24**	B	R	Lt	R			B	R	Lt	R		
**25**			R	R	Lt				R	R	Lt	
**26**		B	Lt	R				R	Lt	R		
**27**			B	R	R				R	R	R	
**28**		B	R	R	B			B	R	R	B	
**29**			Lt	Lt	Lt				Lt	Lt	Lt	
**30**		B	R	R	Lt			B	R	R	Lt	
**31**		Lt	R	B	R			Lt	R	R	R	
**32**		Lt	Lt	B	R			Lt	Lt	Lt	R	
**33**			Lt	R	B				Lt	R	R	
**34**		Lt	R	B	Lt			Lt	R	Lt	Lt	
**35**		Lt	Lt	R	R			Lt	Lt	R	R	

B = Bilateral, L = Lumbar, S = Sacral, Lt = Left, R = Right, and, PN Ant Br = Pelvic Nerve’s Anterior Vesical Branch

### Laterality determined from left and right stimulation of the pelvic nerve’s anterior vesical branch

Maximum detrusor contraction during left and right stimulation of the pelvic nerve’s anterior vesical branch from 19 animals were measured and recorded (Fig 3) (6 of which were part of the cohort presented in Table 2). Using 25% difference, we found that 42% of the animals had left side dominance (n = 8), 32% of the animals had right side dominance (n = 6), and the remaining 26% of the animals were bilateral (n = 5). When the laterality definition was redefined using ± 10%, 42% of the animals had left side dominance (n = 8), 37% of the animals had right side dominance (n = 7), and the remaining 21% of the animals were bilateral (n = 4, Table 1).

## Discussion

Prior to this study, lateralization of bladder function in normal dogs has not been reported. Here, we tested the proportion of contribution of left and right spinal roots from L6 to S3, as well as pelvic nerve’s anterior vesical branch, to bladder contraction using electrical stimulation of these structures in normal female dogs. These data suggest lateralization of bladder function in approximately 2/3 of the animals when using a > 25% difference in maximum detrusor pressure across all levels, when one side was stimulated versus the opposite side. More specifically, about 1/3 of the animals had lateralization to the right, 1/3 to the left, and the remaining 1/3 had bilateral contribution to the functional innervation of the bladder. When lateralization was instead defined as a >10% difference between left and right, a lower propostion of the animals were considered bilateral but there was not any greater proportion considered left or right side dominance.

Interestingly, lateralization was not exclusive to one side across the population or an individual spinal level. This observation is important to consider when addressing previous studies, which attempted bladder reinnervation for a variety of pathologies including spinal cord injury and spina bifida using a somatic-central nervous system-autonomic reflex pathway via L5 ventral root anastomosis to the S3 ventral root exclusively on the left side [12, 13]. To date, no evidence of left-sided laterality has been reported for the human bladder. A retrograde neurotracing study in the rat found that neurons in the major pelvic ganglion, pelvic accessory ganglion and dorsal root ganglia were retrogradely labeled from the ipsilateral bladder (50–65%), the contralateral bladder (31–41%) and from both sides 4–8%, but no comparison of left side compared to right side labeling was reported [17]. With emerging interventions such as surgical reinnervation of the bladder, the possibility of heterogeneity in lateralization and the possible benefit of a bilateral approach to reinnervation should be considered.

## Conclusion

Overall, these data provide evidence for lateralization of the functional innervation of the bladder across normal female dogs; however, left- and right-sided lateralization occurred at similar rates and lateralization often varied per spinal cord level within the same animal. In addition, some dogs had bilateral distribution of functional innervation. Although, the current observations were obtained from normal dogs, developing a means to determine lateralization of functional innervation of the bladder may be important to understanding the consequences of lateralization in patients with bladder dysfunctions.
